# Supplementary figures and images for: New Coronavirus in Colombian Caribbean Bats: In Silico Analysis Reveals Possible Risk of Interspecific Jumping
Source: Viruses. 2025 Sep 29;17(10):1320. doi: 10.3390/v17101320 (PMC12568244; doi:10.3390/v17101320)

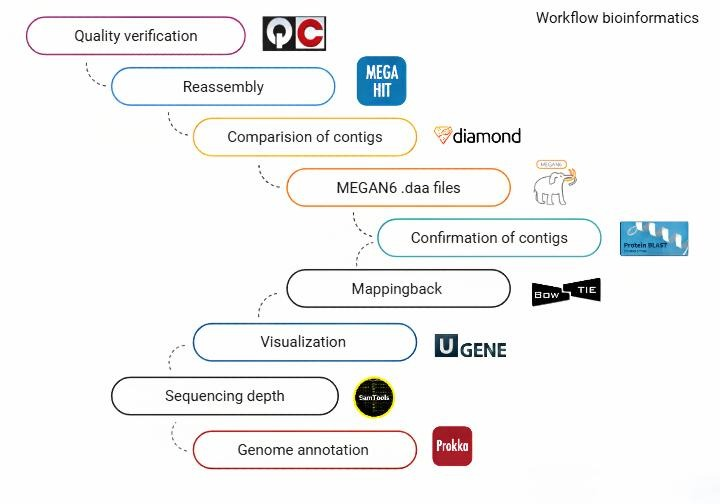

Supplement: Supplementary file 1 [file viruses-17-01320-s001.zip › Figure S1 Bioinformatic workflow for genome assembly and phylogenetic analysis.tiff]

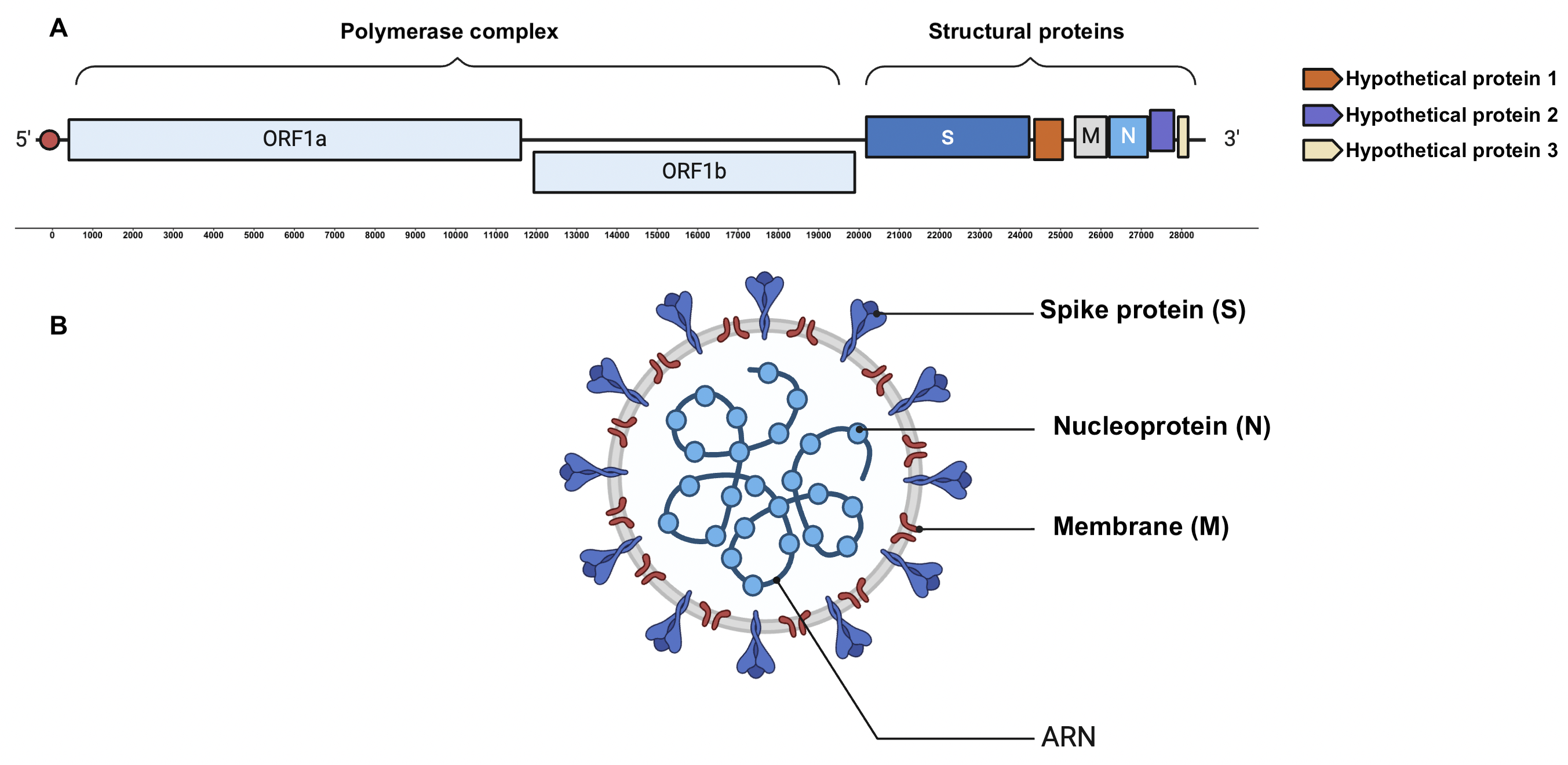

Supplement: Supplementary file 1 [file viruses-17-01320-s001.zip › S2-Genome_Supplementary.tiff]

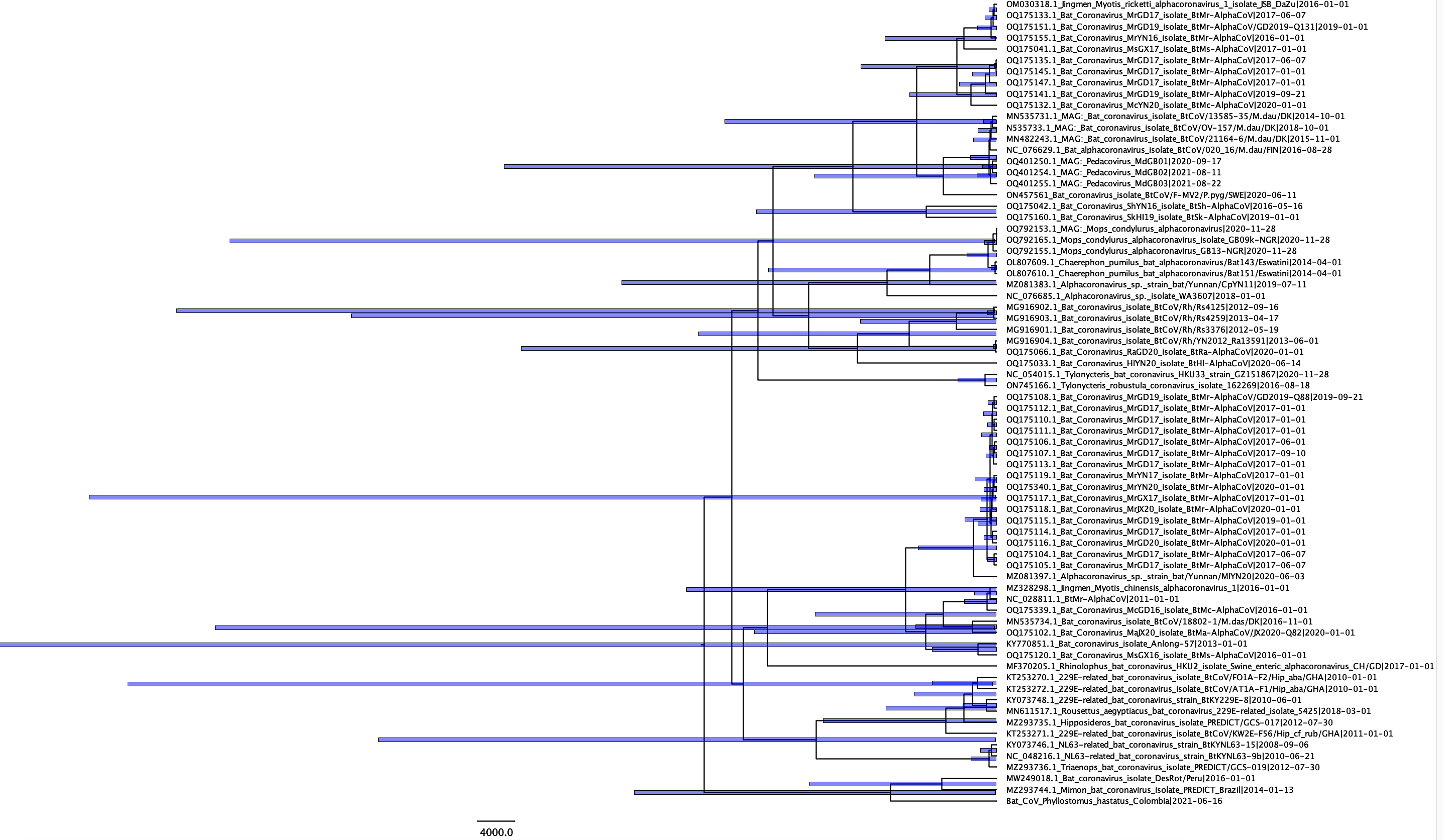

Supplement: Supplementary file 1 [file viruses-17-01320-s001.zip › S3-TMCR_Supplementary.tiff]

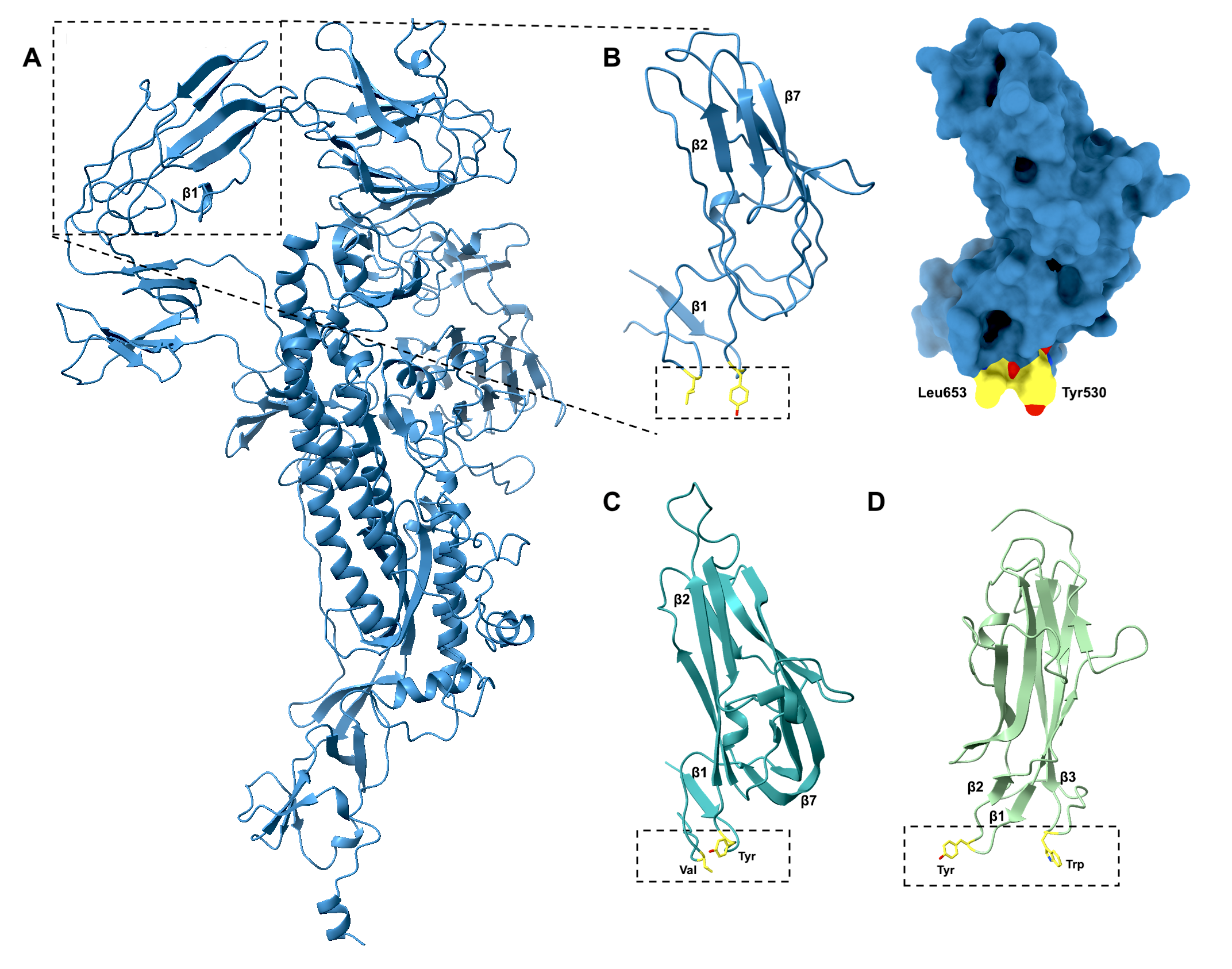

Supplement: Supplementary file 1 [file viruses-17-01320-s001.zip › S5-RBD_Supplementary.tiff]
